# Assessment of male reproductive traits in endangered leuciscids from the Iberian Peninsula: first attempts to store gametes both at short- and long-term

**DOI:** 10.1007/s10695-023-01195-4

**Published:** 2023-04-21

**Authors:** Ana Hernández-Rodríguez, Carla Sousa-Santos, Fátima Gil, Elsa Cabrita, Pedro M. Guerreiro, Victor Gallego

**Affiliations:** 1 Centre of Marine Sciences (CCMAR), University of Algarve, Faro, Portugal; 2 MARE - Marine and Environmental Sciences Centre, ISPA, Lisbon, Portugal; 3 Aquário Vasco da Gama, Marinha Portuguesa, Lisbon, Portugal

**Keywords:** Leuciscidae, Sperm motility, Short-term storage, Sperm cryopreservation

## Abstract

During the spring of 2022, several endangered leuciscid species (*Anaecypris hispanica*, *Squalius aradensis*, *Anachondrostoma Occidentale*, and *Iberochondrostoma lusitanicum*) were sampled both at the Vasco da Gama aquarium facilities and in some rivers of the Algarve region, Portugal. Sperm samples were extracted by gentle abdominal pressure and sperm motion parameters were assessed for the first time in four species, using a computerized analysis system. The results obtained showed that spermatozoa kinetic patterns were similar for all 4 species, with high motility and velocity values after the sperm activation time and with a marked decrease after 20. On the other hand, sperm longevity was highly variable between species, with short longevities (around 40 s) for *A. hispanica* and *S. aradensis*, and longer longevities (100–120 s) for *A. occidentale* and *I. lusitanicum*, which could indicate a latitudinal pattern in terms of sperm longevity*.* At the same time, morphometric analysis was carried out for the four target species, revealing that spermatozoa showed similar sizes and shapes to other external fertilizers belonging to Leuscididae, with small spherical heads, uniflagellate, and without acrosomes. In addition, a short-term gamete storage trail was performed by diluting sperm in 1:9 (sperm:extender) and storing them at 4ºC. Although the results obtained were uneven among the species studied, the dilution and extender used generated motilities above 40% up to day 4 of storage in *S. aradensis* and *I. lusitanicum*, and up to days 1–2 in *A. hispanica* and *A. occidentale*, respectively. Finally, gamete cryopreservation trials were also carried out on these threatened species. Although cryopreserved samples showed significantly lower motility than fresh samples, some protocols generate acceptable percentages of viability, DNA integrity, and sperm motility in some species such as *I. lusitanicum* and *A. occidentale*. The data revealed that the protocol based on 10% DMSO plus 7.5% egg yolk generated the best results.

This study is the first to assess the reproductive traits of wild and captive populations of endangered leuciscids endemic from the Iberian Peninsula, describing the spermatozoa kinetics and developing protocols for managing male gametes both in short- and long-term storage. Outcomes will provide new and useful tools to complement the management and conservation of ex situ breeding programs that are being developed for these four endangered species.

**Supplementary Information:**

The online version contains supplementary material available at 10.1007/s10695-023-01195-4.

## Introduction

Populations of fish species endemic of the Iberian Peninsula have been declining since the mid-twentieth century due to factors such as habitat degradation and fragmentation, dredging and draining processes or water quality deterioration (Antunes et al. 2015). In addition to these factors, the typical summer droughts that impose mass killings and genetic bottle necks are an additional extinction risk factor for the Iberian native freshwater species, especially in Mediterranean-type southern streams. With the tendency for global warming in Iberia, these droughts show consistent increase in duration and severity, raising the pressure and the risk of local extinctions (Ribeiro et al. 2020). In this regard, from the 35 Iberian native species belonging to *cyprinidae* and *leuciscidae*, around 70% are already under threat according to International Union for Conservation of Nature (IUCN 2022). This is the case of the target species of that study: the vulnerable Arade chub (*Squalius aradensis*), the endangered Saramugo (*Anaecypris hispanica*), the endangered Western ruivaco (*Achondrostoma occidentale*), and the critically endangered Portuguese arched-mouth nase (*Iberochondrostoma lusitanicum*). Although they are mostly small leuciscids with little or no economic value, they play a key role in the aquatic ecosystem, and they are important for biodiversity conservation.

Several types of actions have been applied over the past decades for preserving these native fish species, and in situ measurements (monitoring programs, ecosystem restoration, etc.) have been successfully complemented with ex situ conservation actions (Maceda-Veiga 2013). Within these achievements, the launch of successful captive breeding programs carried out by the academia, governmental agencies, and non-governmental organizations, allowed to support these several critically endangered Iberian endemic fish (Sousa-Santos et al. 2014). These ex situ reproductive programs were able to partially recover natural populations in eminent risk of extinction, by periodically releasing fish and reinforcing the numbers of specific populations or recreating population nuclei restored habitats. Furthermore, the scarce knowledge on the reproductive biology of these species highlights the need to investigate different aspects of their reproductive cycles, such as the duration of the reproductive period, the study of gamete quality, or the possibility for long-term conservation of gametes. In this sense, during the last few years there has been much attention on the development of reproduction biotechnology of rheophilic fish belonging to the family *Leuciscidae* (Cejko et al. 2011; Fernández‐Delgado and Herrera 1995; Kowalski et al. 2012; Pires et al. 2000). Thus, the evaluation of breeders throughout their annual cycle is an indispensable tool to obtain an acceptable yield in terms of larval and fry production, which can be used in the future to repopulate certain areas where these species can be usually found.

Following the same line of conservation approach and complementing the in situ and ex situ preservation efforts, a strategy currently applied in fish management is the creation of genetic resource banks (GRBs) (Martínez-Páramo et al. 2017). GRBs are usually based on cryopreservation techniques, which aim for placing and holding the biological material at ultra-low temperatures for a long period. In that way, the use of GRBs for captive breeding programs make possible to develop several tasks, including (i) the preservation of the genetic material of endangered species, (ii) the conservation of certain genetic lines or haplotypes within the same species, or (iii) the recovery of lost genetic characteristics in some populations or individuals (becoming a wild-phenotypic backup). Even though GBRs are being used systematically in various programs aimed at the conservation of large mammals (felines, canids, or bears), their role as a conservation tool in fish is practically focused on aquaculture production (Mayer 2019). To date, sperm of more than 200 fish species have been successfully cryopreserved and cryo-protocols for sperm management have been established for freshwater and marine fish species (Betsy and Kumar 2020). Nevertheless, most of the studies show a great variability between species, populations, individuals from the same population, and even between samples from the same individuals (Blanes-García et al. 2021). Therefore, the design of a cryopreservation protocol must be a meticulous (species-specific) task that should consider the specific characteristics of the gametes of each species to determine the most appropriate parameters for optimizing the cryopreservation process. In the case of species from the family Leuciscidae, some attempts have been successfully applied both short- and long-term gamete storage in species such as the *Leuciscus idus* (Bernáth et al. 2018; Cejko et al. 2022).

Following this rationale, this work attempts, as a complement to the ex situ conservation actions, to advance in the reproductive biology and cryobiology of some endangered species belonging to the family Leusciscidae that are catalogued in different degrees of vulnerability according to the IUCN Red List. The output knowledge will help in the future management and conservation of the endangered ichthyofauna of the Iberian Peninsula.

## Methods

### Ethics statement

This study was carried out in strict accordance with the recommendations given in the Guide for the Care and Use of Laboratory Animals of the EU Directive 2010/63/UE, transposed in the Portuguese legislation by the Decree-Law 113/2013, with modifications included in decree-law 1/2019, regarding the protection of animals used for scientific purposes. The protocol was approved by the Experimental Animal Ethics Committee from the CCMAR (University of Algarve) and permits for field work were given by ICNF (Fish capture and handling licenses: 515/2022/CAPT (CSS), 509/2022/CAPT (VGA) and 506/2022/CAPT (PMG).

### Fish sampling

To obtain gamete samples of the target species (*A. hispanica*, *S. aradensis*, *A. occidentale*, and *I. lusitanicum*), several samplings were carried out during the months of March, April, and May 2022 both at the Vasco da Gama Aquarium (AVG, Lisbon, Portugal) and in different rivers of the Algarve region, Portugal. Sampling at the aquarium was carried out during tank cleaning days, as individuals had to be taken out for annual monitoring of body condition. During these working days, the specimens of *A. hispanica*, *A. occidentale*, and *I. lusitanicum* were removed from their tanks, weighted, measured, and the male individuals were separated for sampling.

As for the sampling carried out at different points in the Algarve region, targeted electric fishing was carried out with the aim of obtaining specimens of the Arade chub (*S. aradensis*). A portable, battery-powered (SUM, Poland) electric fishing equipment was used, carrying out zigzag transects of 15–20 min against the current, searching for specimens among the rocks and vegetation. The captured stunned specimens were removed from the environment and, after obtaining the gamete sample, were allowed to recover and returned live to their natural habitat.

### Collection of sperm samples

For the collection of sperm, the genital area was cleaned with 1% NaCl (pH = 8.0, osmolarity = 300 mOsm/kg) and dried to avoid contamination by faeces, urine, or tank water that could activate the sperm. Using the abdominal massage technique performed with two fingers, gamete samples were collected. The number of sperm samples obtained was different for each species: 12 for *A. hispanica*, 10 for *S. aradensis*, 52 for *A. occidentale*, and 27 for *I. lusitanicum*. The small amount of sperm obtained (which varied according to the species) was collected with a pipette (P-100) and deposited in 500 µl microtubes. The volume of each sample was assessed, and then the sperm was diluted 1:9 (sperm:extender) with SMIS solution (Sperm motility-inhibiting saline solution). This medium has been used previously in other leuciscid species as an extender (Lahnsteiner and Mansour 2010) and it is composed with 75 mmol/L NaCl, 70 mmol/L KCl, 2 mmol/L CaCl_2_, 1 mmol/L MgSO_4_, and 20 mmol/L Tris. Finally, microtubes with sperm samples were kept in a portable fridge (4 ºC) up to 2–3 h, until the sperm analyses were carried out at laboratory facilities. To measure sperm density, samples were previously diluted in SMIS medium, and finally the dilution was taken for counting the spermatozoa in a hemacytometer.

### Assessment of sperm motion parameters

The sperm samples (previously diluted 1:9) were activated by mixing 0.5 μl of diluted sperm with 4.5 μL of freshwater of their environments (0–50 mOsm/kg, pH = 7.0–8.0) in a Makler Chamber (10-μm depth). Samples were analyzed with ISAS (Integrated Semen Analysis System) software, capturing 0.5-s video sequences at 25 fps (frames per second) using a camera attached to a phase contrast microscope (Nikon Eclipse E200) through 10 × lens. All the motility analyses were performed in triplicate. Sperm samples with motility above 60% were selected for characterized the sperm motion parameters and kinetic patterns of each species: 7 for *A. hispanica*, 7 for *S. aradensis*, 11 for *A. occidentale*, and 13 for *I. lusitanicum*.

The parameters used in this study were total motility (MOT, %), defined as the percentage of motile cells in the sample; progressive motility (pMOT, %), defined as the percentage of spermatozoa moving essentially in a straight line; curvilinear velocity (VCL, μm/s), defined as the total distance travelled by the sperm head per unit time; the rectilinear velocity (VSL, μm/s), determined from the straight-line distance between the first and last point of its trajectory; and the percentage of fast (VAP > 100 μm/s), medium (VAP = 50–100 μm/s) and slow (VAP = 10–50 μm/s) spermatozoa, where VAP (mean trajectory velocity), is defined as the distance travelled by the spermatozoa along the mean trajectory. Sperm samples were considered motile if their total motility was over 10%.

### Study of morphological parameters

Sperm samples were fixed in 1% glutaraldehyde for subsequent analysis with a phase contrast optical microscope (Nikon Eclipse E200) using a 100 × lens. Photographs of 50 spermatozoa from 5 individuals for each species were taken with a camera (VisiCam 16 Plus VWR) connected to the microscope. The images obtained were analyzed with ImageJ software, and measurements of the sperm head and flagella were taken. Regarding spermatozoa head measurements, several size variables such as length (L, µm), width (W, µm), area (A, µm2), and perimeter (P, µm) and shape variables such as ellipticity (L/W) were also measured.

### Short- and long-term preservation protocols

Sperm samples with motility above 60% were selected for carrying out the short-term preservation trial. Sperm samples were stored in the refrigerator (4 °C) diluted at 1:9 (sperm:SMIS), with a final concentration ranging from 3 to 5 × 10^8^ spz/mL in *A. hispanica*, 1–2 × 10^8^ spz/mL in *S. aradensis*, 6–8 × 10^8^ spz/mL in *A. occidentale*, and 5–6 × 10^8^ spz/mL in *I. lusitanicum*. Samples were stored in 500-µl microtubes (closed) without shaking, and they were analyzed every 24 h with ISAS software (see the “Collection of sperm samples” 2.3) until their total motility dropped below 5%.

Regarding cryopreservation trials, sperm samples with motility greater than 60% (for *A. hispanica* and *I. lusitanicum*), 50% (for *A. occidentale*), and 40% (for *S. aradensis*) were selected for cryopreservation assays. Samples were diluted 1:8 (sperm:extender) in a SIMS solution and different concentrations (from 5 to 10%) of internal (methanol, MET; dimethyl sulfoxide, DMSO; and glycerol, GLY) and external (egg yolk, EY) cryoprotectants were tested. Final concentrations of sperm samples before freezing ranged from 3 to 5 × 10^8^ spz/mL in *A. hispanica*, 1–2 × 10^8^ spz/mL in *S. aradensis*, 6–8 × 10^8^ spz/mL in *A. occidentale*, and 5–6 × 10^8^ spz/mL in *I. lusitanicum*. Samples were immediately packed in straws of 250 µl. Between two and three straws from each specimen were used, according to the available sperm volume. The diluted samples were then incubated for 5 min at 4 °C. The freezing condition was created by placing the straws on a floating structure 2 cm over the liquid nitrogen (LN) for 5 min in a polystyrene box, and then by dropping them into the LN. For the thawing process, the straws were thawed at 25 °C for 15 s. The motility patterns were again analyzed by triplicate using the ISAS software (see the “Collection of sperm samples” section), and further analyses were carried out for assessing the gamete viability and the DNA fragmentation.

### Gamete viability

Cell viability was conducted for the cryopreservation trial in every fresh and thawed sample using a fluorescence kit (LIVE/DEAD Sperm Viability Kit, Thermo Fisher Scientific, MA, USA) containing SYBR 14 and propidium iodide (PI). For each sample, previously diluted at 1:200 (sperm:extender), 0.5 µL of SYBR 14 (final concentration 100 nM) and 2 µL of PI (final concentration 12 µM) were added to 50 µL of fresh or thawed sperm samples and incubated at room temperature and darkness for 10 min. Thereafter, each sample was placed on a slide for analysis in a fluorescence microscope. Photographs of fresh and thawed samples were taken with a VisiCam 16 Plus VWR camera at 20 × magnification. Then 100 cells were counted using ImageJ software and the number of live cells (green) and dead cells (red) was calculated.

### DNA fragmentation (comet assay)

The protocol used was developed by Cabrita et al. (2005) with some modifications to fit the target species. Agarose coated slides were used to support 50 µl of cells previously embedded in low-melting agarose. After incubation in a lysis solution and electrophoresis, the samples were incubated in a neutralization solution and finally fixed with ethanol. Once dried and fixed, the samples were stained with PI and observed with a 40 × objective on a fluorescence microscope. Photographs were taken with Wavelmage software and then cell damage was analyzed with Komet6 brand. Measurements of 100 cells per male and treatment were taken to compare the cell damage of fresh and cryopreserved samples. The parameter analyzed was the percentage of DNA in the tail (% DNAt).

### Statistical analysis

The mean ± standard error was calculated for all sperm quantity and quality parameters. Shapiro–Wilk and Levene tests were used to check the normality of data distribution and variance homogeneity, respectively. For non-normal distributions of percentage values, the data were transformed using arc-sin transformation prior to statistical analysis. Univariate General Linear Model (GLM) and Student–Newman–Keuls (SNK) tests were used to analyze the sperm kinetic parameters along post-activation times. One-way ANOVA was used to analyze the morphometric parameters and the different cryopreservation protocols. Significant differences were detected when *p-*value < 0.05. All statistical analyses were performed using the statistical package SPSS version 24.0 for Windows software (SPSS Inc., Chicago, IL, USA).

## Results

### Sperm density, volume, and motility

**Fig. 1:**
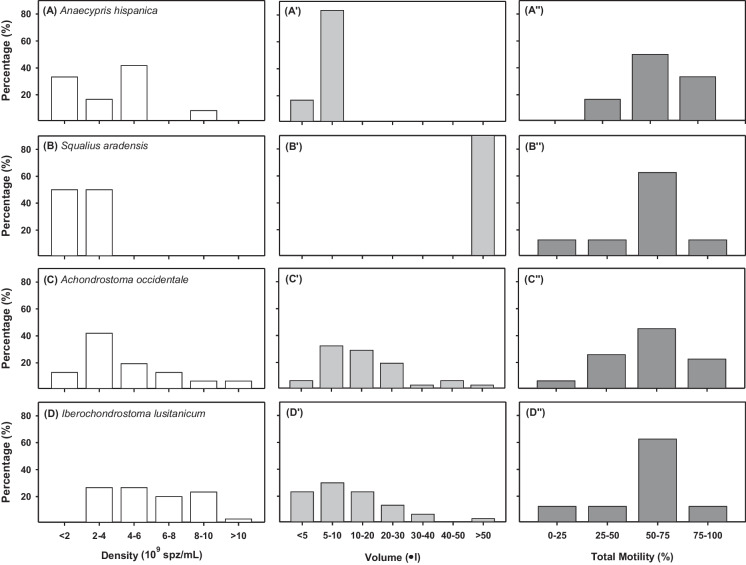
Histograms of the sperm density, sperm volume and sperm motility of **A** *A. hispanica* (*n* = 12), **B*** S. aradensis* (*n* = 8), **C** *A. occidentale* (*n* = 30), and **D** *I. lusitanicum* (*n* = 31)

The samples obtained showed a wide range of spermatozoa densities among the species studied (Fig. 1; and Supplementary Data), ranging from 5 × 10^8^ spz/mL in *A. hispanica* to more than 1 × 10^10^ in the case of *A. occidentale* (Fig. 1A and 1C, respectively). In all samples of *S. aradensis* the density values were between 1 and 4 × 10^9^ spz/mL, while in the rest of the species the variability was much higher. In terms of volume, *A. occidentale* and *I. lusitanicum* showed a similar distribution with a very wide volume range, from less than 5 μL to more than 50 μL per individual (Fig. 1C', D'). *A. hispanica* individuals generated the smallest volumes (Fig. 1A'), with most samples (~ 80%) presenting volumes between 5 and 10 μL. In contrast, *S. aradensis* individuals showed high sperm volumes (Fig. 1B') and all samples collected were between 100 and 460 μL (Supplementary Data). The histograms obtained for the sperm motility were similar for all species, with a relatively high percentage of males (40–60%) showing good quality samples (between 50 and 75% of motile cells), and 10–30% of males showing high quality sperm samples (motility values over 75%).

All the statistical values (mean; standard deviation; standard error; minimum and maximum values; and lower and upper confidence intervals at 95%) regarding sperm density, volume, and motility can be consulted in Supplementary Material.

### Sperm motion parameters over time

**Fig. 2:**
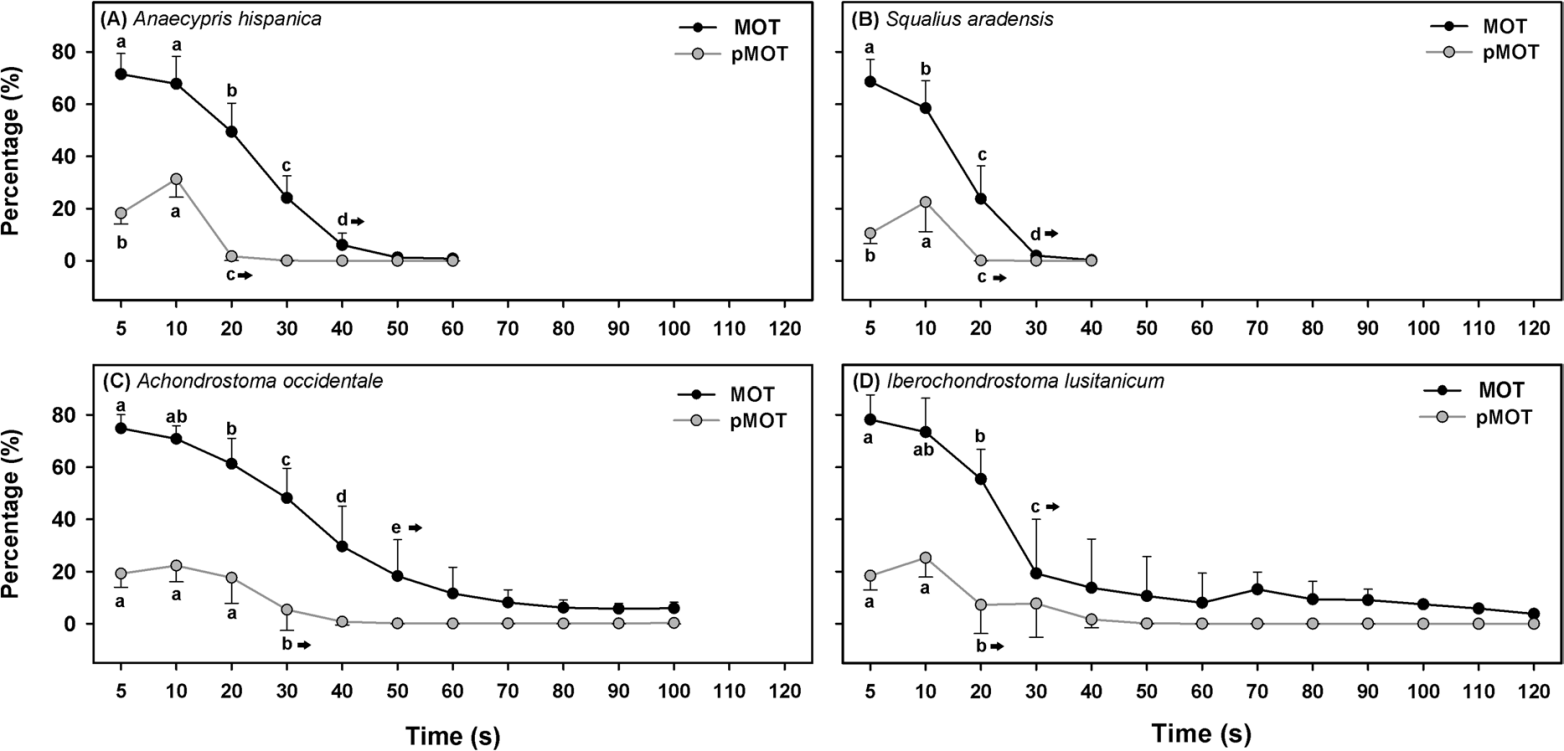
Total (MOT) and progressive (pMOT) motility of **A** *A. hispanica* (*n* = 7), **B** *S. aradensis* (*n* = 7), **C** *A. occidentale* (*n* = 11), and **D** *I. lusitanicum* (*n* = 13) at different post activation times Data are expressed as mean ± standard error. Letters indicate significant differences between post-activation times

All four species showed high motility (around 70–80%) during the first 5–10 s post-activation, with a marked and significant decrease at 20 s in all species (Fig. 2). The motility patterns were very similar in *A. hispanica* and *S. aradensis*, while the pattern of *A. occidentale* was similar to *I. lusitanicum*. The most notable difference in the sperm motion parameters was in the swimming time: the species with the longest sperm duration was *I. lusitanicum* (120 s) and the species with the shortest longevity was *S. aradensis* (40 s).

**Fig. 3:**
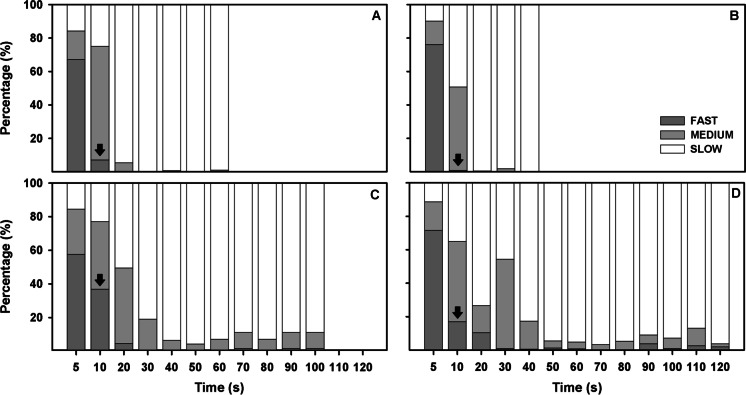
Percentage of fast, medium, and slow spermatozoa in **A** *A. hispanica* (*n* = 7), **B*** S. aradensis* (*n* = 7), **C** *A. occidentale* (*n* = 11), and **D** *I. lusitanicum* (*n* = 13) at different post activation times. Arrows indicate the first significant difference on fast spermatozoa from the sperm activation point (5 s)

Regarding sperm kinetics (Fig. 3), all four species showed a high percentage of fast spermatozoa (> 60%) during the first 5 s post-activation. However, from 10 s post-activation, the percentage of fast spermatozoa decreased to less than 30% in the case of *A. occidentale* and *I. lusitanicum*; and to less than 10% in *A. hispanica* and *S. aradensis*.

### Sperm morphology

**Fig. 4:**
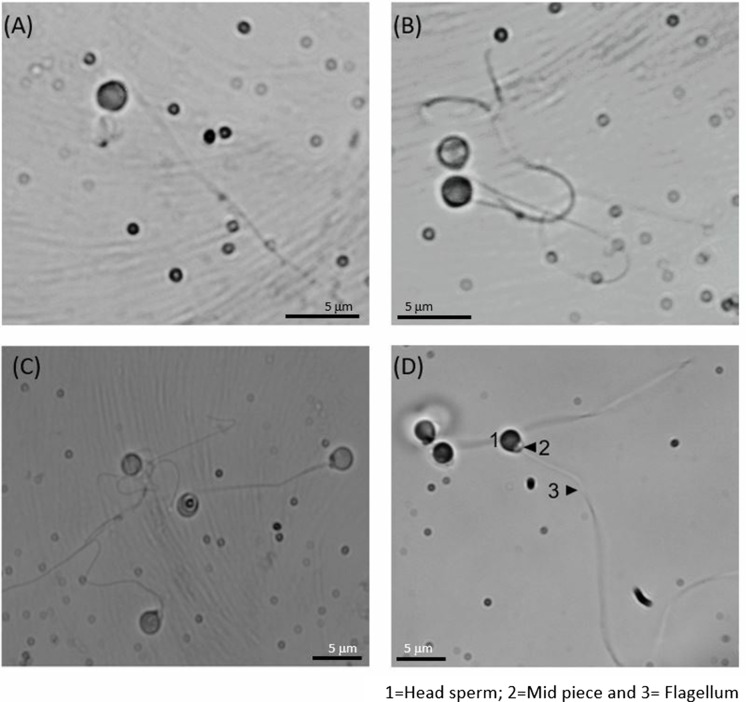
Spermatozoa morphology of **A** *A. hispanica* **B** *S. aradensis*, **C** *A. occidentale*, and **D** *I. lusitanicum*. Pictures A and B were taken using the 100 × lens and pictures **C** and **D** using the 40 × lens. Scale bar is showed in the pictures

The different morphometric parameters studied using ImageJ software showed significant differences between the species studied (Table 1). The species with the largest spermatozoa (head and flagellum) was *A. occidentale*, with significant differences in almost all morphometric parameters compared to the other 3 species. *A. hispanica* and *S. aradensis* had the smallest spermatozoa, with no marked differences between them but with significant differences compared to the other two species (*A. occidentale* and *I. lusitanicum*). Figure 4 shows optical microscope photographs (100 ×) of the spermatozoa of all species studied. All of them showed a similar structure with a spherical head (1), a middle part (2), and a flagellum (3).

**Table 1:** Morphometric parameters of the head and flagellum of spermatozoa of *A. hispanica*, *S. aradensis*, *A. occidentale*, and *I. lusitanicum* measured with ImageJ software using a light microscopy (*n* = 50 spermatozoa of 5 specimens). Data are expressed as mean ± standard error and letters mean significant differences between species

	*A. hispanica*	*S. aradensis*	*A. occidentale*	*I. lusitanicum*
Area (µm^2^)	3.83 ± 0.06 **c**	3.63 ± 0.06 **c**	5.13 ± 0.03 **a**	4.85 ± 0.10 **b**
Perimeter (µm)	9.84 ± 0.18 **c**	9.41 ± 0.19 **c**	13.88 ± 0.41 **a**	11.06 ± 0.12 **b**
Width (µm)	2.00 ± 0.00 **b**	2.00 ± 0.00 **b**	2.21 ± 0.03 **a**	2.08 ± 0.03 **b**
Length (µm)	2.04 ± 0.02 **b**	2.04 ± 0.01 **b**	2.90 ± 0.02 **a**	2.83 ± 0.07 **a**
Ellipticity	0.93 ± 0.01 **a**	0.93 ± 0.00 **a**	0.90 ± 0.01 **b**	0.90 ± 0.00 **b**
Flagellum	26.4 ± 3.7 **b**	23.1 ± 1.2** c**	31.1 ± 1.5 **a**	28.6 ± 1.7 **ab**

### Short-term gamete preservation

**Fig. 5:**
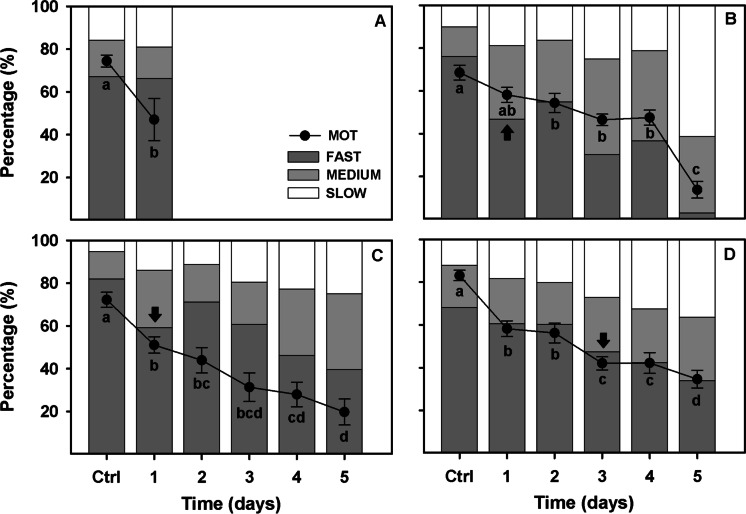
Percentage of total motility (dot lines) and the fast, medium, and slow spermatozoa over storage time (1 to 5 days) in **A** *A. hispanica* (*n* = 7), **B*** S. aradensis* (*n* = 7), **C** *A. occidentale* (*n* = 11)*,* and **D** *I. lusitanicum* (*n* = 13), at different post activation times. Data are expressed as mean ± standard error. Letters indicate significant differences between post activation times; and arrows indicates the first significant difference on fast spermatozoa from the sperm activation point (5 s)

The sperm quality of samples diluted (1:9 in SMIS) and stored at 4 °C was studied over 5 days of storage (Fig. 5). In relation to sperm motility, 3 of 4 species studied (*A. hispanica*, *A. occidentale*, and *I. lusitanicum*) suffered a significant decrease in the percentage of motile spermatozoa after 24 h of storage. However, in some species such as *S. aradensis* and *I. lusitanicum*, sperm motility did not decrease drastically over time, even presenting values of more than 40% of motile spermatozoa at 4 days of storage (Fig. 5B, D).

In relation to velocities (fast, medium, and slow spermatozoa), the results showed a progressive drop in the number of fast spermatozoa over time in all species (and therefore an increase in the percentage of slow and medium spermatozoa). However, this drop was very marked in species such as *S. aradensis* and *A. occidentale* (with significant differences on day 1) and very mild in species such as *I. lusitanicum*, which showed a significantly lower percentage of fast spermatozoa on day 3 of storage.

Finally, it is important to note that in the case of *A. hispanica* it was not possible to study short-term preservation for more than two days, as due to the small volume generated by this species there was not enough sample volume for a continuous evaluation.

### Cryopreservation trials

Results from the cryopreservation trials showed that the cryopreservation process caused a reduction of the sperm viability and motility parameters (irrespective of the protocol used) in all the species studied (Table 2). In the case of *A. hispanica*, protocols based on 10% methanol and DMSO) generated significant differences in viability (from 70% in the control group to 20% in cryopreserved samples), DNA fragmentation (comet assay) and motility (with values from 75% in the control group to 1–3% in cryopreserved samples). In the case of *I. lusitanicum*, although all the protocols tested also significantly decreased the gamete quality, the addition of 7.5% egg yolk to a 10% DMSO-based protocol significantly improved the motility compared to the other protocols used. In this case, the 10% DMSO plus 7.5% egg yolk generated motility values of 15–20%, which was the highest value in all protocols and species studied during the cryopreservation trials. In the case of *S. aradensis* and considering that the initial samples did not show high motility values (45–50%), the protocols tested did not generated good cell viability and motility values. Finally, regarding *A. occidentale*, it was again observed that the addition of egg yolk to a protocol based on 10% DMSO significantly improved motility with respect to the rest of the protocols used. In this case, the protocol based on 10% DMSO plus 7.5% egg yolk produced motility values of 9.2% and viabilities of 24.5%.

**Table 2:** The effects of the different gamete cryopreservation protocols applied in *A. hispanica* (*n* = 5), *S. aradensis* (*n* = 5), *A. occidentale* (*n* = 7), and *I. lusitanicum* (*n* = 9) on viability, DNA fragmentation and spermatozoa motility. Letters indicate significant differences between the protocols used (mean values ± SEM)

Species	Protocol	Viability	SEM	DNA frag	SEM	Motility	SEM
*A. hispanica*	Control	73.6 **a**	8.6	15.3 **a**	0.7	74.4 **a**	2.8
	DMSO-10	23.3 **b**	2.1	26.5 **b**	0.4	3.5 **b**	3.0
	METH-10	20.5 **b**	4.5	29.3 **b**	3.7	1.5 **b**	0.8
*I. lusitanicum*	Control	65.6 **a**	4.4	18.5 **a**	1.0	83.1 **a**	2.4
	DMSO-10	11.9 **c**	1.9	24.3 **b**	1.4	1.0 **c**	0.4
	METH-10	23.6 **b**	2.5	18.5 **a**	1.8	1.4 **c**	0.2
	DMSO-10 + EGG-7.5	33.0 **b**	2.5	16.0 **a**	1.4	16.8 **b**	2.5
	EGG-15	25.9 **b**	4.2	20.1 **a**	1.5	0.2 **c**	0.1
*S. aradensis*	Control	45.0 **a**	4.9	17.1 **a**	0.6	47.5 **a**	2.4
	DMSO-5	10.0 **b**	4.3	20.3 **a**	0.7	0.6 **b**	0.1
	DMSO-10	19.5 **b**	5.7	15.5 **a**	0.0	1.1 **b**	0.2
	DMSO-10 + EGG-10	8.2 **b**	1.9	20.2 **a**	1.2	2.1 **b**	0.7
*A. occidentale*	Control	75.7 **a**	4.6	19.1 **a**	0.9	56.7 **a**	6.5
	DMSO-5	24.7 **b**	6.3	17.6 **a**	1.4	2.0 **c**	0.5
	DMSO-5 + EGG-10	12.6 **c**	1.4	17.4 **a**	1.8	2.2 **c**	0.7
	DMSO-10 + EGG-7.5	24.7 **b**	6.3	18.6 **a**	1.4	9.2 **b**	0.9
	GLY-5	11.3 **c**	3.8	18.6 **a**	2.9	1.1 **c**	0.3
	GLY-5 + EGG-10	7.5 **c**	0.5	20.9 **a**	5.0	1.1 **c**	0.2
	GLY-15	7.0 **c**	0.0	29.2 **b**	0.0	0.0 **c**	0.0

## Discussion

### Sperm quantity and quality

One of the factors to be considered when carrying out ex situ conservation programmes is the quantity and quality of the gametes produced by the breeders. During this work, in the four species studied, the sperm density values obtained ranged from 2 × 10^9^ to 1 × 10^10^ spz/mL. These results are in line with those obtained by other authors in species belonging to the family Leuciscidae in Central Europe and North America. For example, densities of 1 × 10^10^ spz/mL were obtained in specimens of *Rhynchocypris percnurus* (Dietrich et al. 2011), sperm densities of 7–13 × 10^9^ spz/mL were reported in *Leuciscus idus* (Cejko et al. 2022); and finally, in individuals of *Squalis cephalus*, densities between 5 and 7 × 10^9^ spz/mL were reported by (Cejko and Krejszeff 2016). Regarding sperm volume, the amount of milt obtained varied considerably among the species. The lowest sperm volume samples were obtained from *A. hispanica* (5–10 µl), followed by *A. occidentale* and *I. lusitanicum* (5–50 µl), and finally *S. aradensis*, which was the species that generated the highest volumes, with some wild showing samples with more than 400 µl. That huge variability found in sperm volumes between species could be explained, on the one hand, by the size (weight and length) of the specimens. In that sense, the lowest volumes were found in *A. hispanica*, which showed tiny mean weights and lengths of 1.1 ± 0.2 g and 48.3 ± 5.3 mm, respectively. At the other extreme we had the wild population *S. aradensis*, which released average sperm volumes of 218.8 ± 45.3 μL but showing weights and lengths of 14.7 ± 4.2 g and 108.7 ± 11.2 mm, respectively. On the other hand, this huge variability in sperm volumes is also reported in the literature linked to the family Leuciscidae. In that sense, some studies reported sperm volumes ranging from 0.5 to 6 µl in *P. promelas* (Hala et al. 2009; Martinovic-Weigelt et al. 2012), around 10 µl in specimens of *E. percnurus* (Dietrich et al. 2011), and ranging from 0.5 to 2.5 ml in *Alburnus alburnus* (Lahnsteiner et al. 1996). Finally, and thinking in aquaculture issues, to solve the problem of having such low volumes in some species of Leuciscidae family (e.g., *A. hispanica*), for further studies it would be interesting to increase the net gamete production (binomial density-volume) applying hormonal treatments for stimulate the spermiation process. In that sense, this strategy has been already applied in other leuciscid species with great results both in *Leuciscus leuciscus* (Cejko et al. 2012) or *Leuciscus cephalus* (Cejko and Krejszeff 2016).

In addition to sperm quantity parameters (density and volume), when referring to the sperm quality the most used parameter has been the spermatozoa motility (Gallego and Asturiano 2018). In this work we present for the first time the sperm kinetic parameters of four leuciscid species (*A. hispanica, S. aradensis, A. occidentale* and *I. lusitanicum*) analyzed using a computerised gamete analysis system (ISAS). In general, for the 4 target species, it was observed that motility and velocity values were high after freshwater activation (5–10 s), with a marked decrease after 20 s. These motion patterns are similar to other freshwater species of closer families, such as cyprinidae (Alavi et al. 2009) or percidae (Alavi et al. 2010). However, there are only few studies of sperm kinetics over time in fish belonging to the family Leuciscidae. In that sense, a study carried out in the common murre (*A. alburnus*), showed that the kinetic pattern was not so similar to the species studied. In that case, the sperm of *A. Alburnus* reached the maximum motility at 45–50 s post-activation and has a longevity of about 5 min (Lahnsteiner et al. 1996). In addition, a study carried out by Bernáth et al. (2018) on the ide (*L. leuciscus*) showed that longevity of sperm movement did not show a significant decline in sperm motion parameters for up to 120 s. Similar results were also published by Kowalski et al. (2012) on the side, which sperm motility ranged from 50 to 40% (without big differences) on during the whole activation time.

Regarding to longevity — or how long spermatozoa move — the values for the species studied are in the range of other freshwater species, which typically show values of less than 2 min (Browne et al. 2015). In our study, sperm samples from *A. hispanica* and *S. aradensis* had much less longevity (40–60 s) than samples from *A. occidentale* and *I. lusitanicum* (100–120 s). The differences found between these species could be due to the reproductive biology of each species, due to sperm kinetic parameters (motility, speed, and longevity) are usually closely linked to the reproductive behaviour of the species (Gallego et al. 2014). In that sense (and searching for answers regarding the spermatozoa kinetic differences between species), it would be also possible the existence of a latitudinal gradient from the north to the south species. In that sense, for the southern species (more affected by the loss of longitudinal connectivity imposed by increasingly longer and more intense droughts), their recruitment during the spawning season could affected the sperm swimming time and other reproductive traits, while in the northern species (not so affected by desertification events), the reproductive traits would not be so affected.

In relation to morphology study, the analyses carried out revealed that the spermatozoa of the four species studied presented a morphology similar to other cyprinids and leuciscids species with external fertilization, with small sized cells, spherical head, a middle piece and uniflagellate (Neznanova 2012). The sperm morphology of other leuciscid and cyprinid species has been characterised, for example, in *E. percnurus*, where the sperm head is 1.88 µm long and 2.03 µm wide (Dietrich et al. 2014) or in species such as *Chondrostoma nasus* and *Rutilus meidingerii* (Fürböck et al., 2010). In the case of cyprinidae species, the sperm head of *B. barbus* was 1.80 µm (Alavi et al. 2009), while in *C. carpio* the sperm head width varied from 1.3 to 2.0 µm (Verma et al., 2009). Therefore, results published by other authors in other phylogenetically close groups are similar in terms of sperm head dimensions.

### Short- and long-term gamete storage

As two main approaches for gamete storage, short-term storage refers to keeping gametes at above zero temperatures, while long‐term storage infers preservation of gametes at below zero temperatures. Short‐term storage, ranging from days to weeks, provides some advantages for aquaculture practice in terms of many useful features (e.g., sex synchronization, shipping of gametes), and can be carried out both in spermatozoa and eggs (Inanan 2020). During this work, we have attempted to preserve male gametes from four endangered leuciscid species from the Iberian Peninsula in the short-term storage. Although the results obtained were uneven among the species studied, dilution (1:9) and the use of SMIS as extender generated motilities higher than 40% up to the 4th day of storage in *S. aradensis* and *I. lusitanicum*. This information represents an important advance in the management of these species, as the handling of broodstock for certain tasks involving in vitro fertilization processes could be reduced, decreasing the stress to which individuals are subjected during sampling. Some trails have been carried out in other leuciscid species such as the ide (*L. leuciscus*), where Sarosiek et al. (2012) reported sperm motilities from 46 to 33% after 1-day storage and reaching 13% after 5-day storage. However, in another trial carried out on the ide more recently, Bernáth et al. (2018) reported significant decreases of chilled storage on sperm motility (from 50 to 5%) just at 2-day storage. In that sense, the differences found between those trials could be explained due the type of extender used (e.g., SMIS versus TLP) and the sperm:extender ratio applied (1:3 versus 1:9). Considering all the trials, seems to be evident that to dilute the sperm is an essential premise for keep the sperm motility as long as possible, always using a proper extender similar to the seminal plasma composition of target species. In other phylogenetically close species such as *R. percnurus*, Short-term storage of semen was successfully applied to secure 50% sperm motility for 9 days in VRT buffer (Dietrich et al. 2015).

Despite the good results obtained in this study, it is important to note that during storage of the samples in the refrigerator, sedimentation occurred in almost all species. S. *aradensis* samples had a strong tendency to form clumps at the bottom of the vials over the days, and the appearance of these clumps was linked to a sharp drop in sperm motility. In that sense, other studies have shown that continuous gentle agitation during storage improves quality parameters during the first days or weeks by preventing the formation of clumps (Herranz-Jusdado et al. 2019). Therefore, in future studies, it would be interesting to test the gentle mixing of samples (using an electronic shaker) for extending the sperm quality and viability over time (Herranz-Jusdado et al. 2019). As complementary methods, another way to improve short-term preservation would be the addition of antibiotics. In that sense, Cejko et al. (2022) retained in ide sperm the motility and fertilization capacity over 14 days at 4 °C diluting the sperm with TLP supplemented with penicillin/streptomycin, regardless of the dilution ratio used.

Regarding long-term (cryopreservation) storage trials, cryopreserved samples of all species showed lower motility than fresh samples, reaching maximum percentages of 15–20% of motile cells after thawing in the best of the protocols. However, the data obtained indicated that, in general, and for all species studied, the best results in kinetic parameters were obtained using a protocol based on 10% DMSO (used as internal cryoprotectant) with 7.5% egg yolk (used as external cryoprotectant). As far as we know, there are only few reports about the gamete cryopreservation of leuciscid species. One of them was carried out in the lake minnow (*E. percnurus*), and the highest motility value of cryopreserved sperm was obtained applying methanol and DMSO as cryoprotectants in Tris-glucose buffer (Dietrich et al. 2015). Other study carried out by Bernáth et al. (2018) on the ide (*L. leuciscus*), showed no significant differences between fresh and cryopreserved sperm using 10% methanol, but the variability between sample were too high (pMOT; fresh sperm: 49 ± 19% versus and cryopreserved sperm: 22 ± 22%). Our results agree with work published by other authors on other cyprinid species. In this regard, cryopreservation of *Chalcalburnus chalcoides* gametes using 10% DMSO combined with 5% egg yolk generated post-thaw motilities of up to 30% (Lahnsteiner et al. 2000). Finally, the use of 10% DMSO with 10% egg yolk in *Aspius aspius* generated values of 25% motile cells after thawing (Babiak et al. 1998). On the other hand, Urbányi et al. (2006) also demonstrated the efficacy of using methanol and DMSO as cryoprotectants in four different cyprinid species (*R. rutilus*, *A. brama*, *B. bjoerkna*, *B. barbus*). However, the use of methanol as a cryoprotectant during this study did not generate good motility and viability values in any of the species. Even so, for further studies it seems interesting to explore the possibility of mixing methanol with external cryoprotectants (e.g., sugars or egg yolk) with the aim to improve the results obtained.

In addition to motility as a bioindicator of sperm quality, different analyses were also performed to assess sperm cell integrity in both fresh and cryopreserved samples: cell viability and the “Comet assay.” Regarding cell viability, the highest value was obtained in *I. lusitanicum* using the 10% DMSO and 7.5% egg yolk protocol with results of 30–35% live cells after cryopreservation. These results are similar to those obtained in other species: with values close to 35% viability in *Salmo salar* (Kommisrud et al. 2020), 30% in *Silurus glanis* (Linhart et al. 2005). On the other hand, the comet assay was used to assess differences in DNA integrity before and after cryopreservation. Cell damage was higher in cryopreserved samples than in fresh samples in species such as *A. hispanica* and *I. lusitanicum*, where both cryoprotectants (DMSO and methanol) generated similar percentages of DNA damage between 20 and 25%. In contrast, cryopreserved samples of *S. aradensis* and *A. occidentale* showed no significant differences compared to control samples using methanol or DMSO as cryoprotectants. However, the use of 5% glycerol as cryoprotectant generated the highest degradation values throughout the study. In other studies, carried out on commercial species, DNA degradation has been observed with similar values (although slightly higher) of around 30% in *Oncorhynchus mykiss* (Cabrita et al. 2005), 35% in *Sparus aurata* (Cabrita et al. 2005) and 38% in *Dicentrarchus labrax* (Zilli et al. 2003). Finally, it is important to note that the storage of gametes of threatened species is a scientific challenge, but it is important not to consider gene banks as static stores of biodiversity (Herraez et al. 2011). In this sense, the design of the first spermatozoa cryopreservation protocols for *A. hispanica*, *S. aradensis*, *I. lusitanicum*, and *A. occidentale* should be considered only as a starting point, with the final goal of optimising and standardising current protocols to generate a gene bank focused on and for the in situ and ex situ conservation of these species.

## Conclusions

To sum up, this study improves our knowledge of the reproductive biology of four endangered leuciscid species from the Iberian Peninsula (*A. hispanica*, *S. aradensis*, *A. occidentale*, and *I. lusitanicum*)*,* by reporting both sperm motion parameters and spermatozoa morphometric features in all of them. In addition, this study is the first of its kind to achieve gamete cryopreservation of these species, developing protocols for managing male gametes both in short- and long-term storage. Outcomes will provide new and useful tools to complement the management and conservation of ex situ breeding programs that are being developed on these four endangered species.

## Supplementary Information

Below is the link to the electronic supplementary material.

Supplementary file1 (DOCX 18 KB)

## Data Availability

CRYO-FISH will follow the general politics of EU (H2020 Work Programme 2018–2020), helping to promote the policy goals of open innovation, open science and open to the world (three O’s), and our DMP identifies just one category of data and materials: “free available data.” There is a central repository of these data and materials on the CRYO-FISH web page and more data are available from the authors upon reasonable request.
