# Supplementary material for: Assessment of male reproductive traits in endangered leuciscids from the Iberian Peninsula: first attempts to store gametes both at short- and long-term
Source: Fish Physiol Biochem. 2023 Apr 21;50(5):1931–45. doi: 10.1007/s10695-023-01195-4 (PMC11576815; doi:10.1007/s10695-023-01195-4)
Supplement: Supplementary file 1 — Supplementary file1 (DOCX 18 KB) [file 10695_2023_1195_MOESM1_ESM.docx]

**Table X (Supplementary Material).** Statistical parameters of the sperm density, sperm volume and sperm motility of *A. hispanica* (n=12), B) *S. aradensis* (n=8)*,* C) *A. occidentale* (n=30)*,* and D) *I. lusitanicum* (n=31).

|  |  | ***Mean*** | ***SD*** | ***SEM*** | ***Min.*** | ***Max.*** | ***LCI*** | ***UCI*** |
| --- | --- | --- | --- | --- | --- | --- | --- | --- |
| ***A. Hispanica*** | *Volume* | *7.0* | *2.0* | *0.6* | *2.0* | *9.0* | *5.7* | *8.3* |
| *(n=12)* | *Density* | *3.6* | *2.4* | *0.7* | *0.6* | *9.0* | *2.1* | *5.1* |
|  | *Motility* | *66.6* | *14.2* | *4.1* | *42.0* | *89.4* | *57.6* | *75.7* |
|  |  |  |  |  |  |  |  |  |
| *S. aradensis* | *Volume* | *218.8* | *127.3* | *45.0* | *100.0* | *460.0* | *112.3* | *325.2* |
| *(n=8)* | *Density* | *1.6* | *0.8* | *0.3* | *0.3* | *2.4* | *1.0* | *2.3* |
|  | *Motility* | *57.2* | *17.0* | *6.0* | *21.3* | *76.0* | *43.0* | *71.5* |
|  |  |  |  |  |  |  |  |  |
| *A. occiddentale* | *Volume* | *21.6* | *13.9* | *2.5* | *5.0* | *60.0* | *16.5* | *26.7* |
| *(n=31)* | *Density* | *4.5* | *2.7* | *0.5* | *0.8* | *11.0* | *3.5* | *5.5* |
|  | *Motility* | *59.6* | *17.2* | *3.1* | *20.1* | *84.6* | *53.4* | *65.9* |
|  |  |  |  |  |  |  |  |  |
| *I. lusitanicum* | *Volume* | *17.5* | *13.2* | *2.4* | *5.0* | *60.0* | *12.6* | *22.5* |
| *(n=30)* | *Density* | *6.1* | *2.5* | *0.5* | *2.2* | *10.1* | *5.1* | *7.0* |
|  | *Motility* | *75.5* | *9.8* | *1.8* | *48.6* | *93.9* | *71.9* | *79.2* |

*SD: Standard Deviation; SEM: Standard Error; Min: Minimum Value; Max: Minimum Value; LCL: Lower Confidence Interval (95%); UCL: LCL: Upper Confidence Interval (95%). Volume (μL); Motility (%); Density (x10^9^ spz/mL).*
